# Uncovering the Targets of Pueraria Associated with Programmed Cell Death and the Construction of a Diagnostic Model in Septic Cardiomyopathy

**DOI:** 10.3390/biomedicines14051114

**Published:** 2026-05-14

**Authors:** Fuwei Liu, Jun Luo, Peng Yu, Jianzhong Zhou

**Affiliations:** 1 Department of Cardiology, The First Affiliated Hospital of Chongqing Medical University, Chongqing 400016, China; gzliufuwei@163.com; 2 Department of Cardiology, The Affiliated Ganzhou Hospital, Jiangxi Medical College, Nanchang University, Ganzhou 341000, China; 3 Department of Endocrinology and Metabolism, The Second Affiliated Hospital of Nanchang University, Nanchang 330006, China

**Keywords:** sepsis, cardiomyopathy, pueraria, cell death, molecular targeted therapy, machine learning

## Abstract

**Background**: Septic cardiomyopathy (SCM) is a fatal sepsis-induced dysfunction. While Pueraria (Pue) exhibits protective effects in sepsis, its regulatory role regarding programmed cell death (PCD) in SCM remains unclear. This study aimed to identify Pue’s PCD-related targets in SCM and construct a validated diagnostic model. **Methods**: We analyzed 14 PCD modalities across seven GEO transcriptomic datasets. A robust machine learning framework integrating 171 algorithm combinations built a diagnostic signature. The immune landscape was profiled using single-cell RNA sequencing and enrichment analyses. Experimental validation utilized SCM patient blood samples and heart tissues from an LPS-induced murine model. **Results**: Nine PCD patterns were significantly altered in SCM. Intersection analysis and machine learning identified five core Pue targets: *STAT3*, *RIPK2*, *GM2A*, *ALOX5*, and *DPP4*. A diagnostic model constructed with these genes achieved high AUCs across all datasets. Single-cell analysis revealed cell-type-specific expression within the myocardial immune landscape. Differential expression of these five genes was validated in both human and animal samples, correlating significantly with cardiac function indices. **Conclusions**: Our results demonstrate that Pueraria mitigates SCM and restores cardiac function by modulating the expression of core PCD-related targets. These targets are closely associated with the localized inflammatory response, providing potential therapeutic avenues for SCM.

## 1. Introduction

The clinical management of septic cardiomyopathy (SCM) remains exceptionally challenging due to an incomplete understanding of its pathogenesis and the absence of standardized diagnostic protocols. As a severe downstream complication of infection-induced systemic inflammation, SCM frequently drives fatal outcomes in patients, deteriorating into multiple organ dysfunction syndrome (MODS), thereby generating substantial global healthcare burdens [1,2,3]. To address this critical therapeutic void, recent research has highlighted the pharmacological promise of Puerarin (Pue). Extracted as the primary bioactive constituent from the traditional botanical remedy *Pueraria lobata*—historically utilized for managing cardiac impairments, emesis, and febrile conditions [4] —this agent has demonstrated profound cardioprotective activity during septic events. Contemporary investigations reveal that Pue actively mitigates cellular injury by dampening inflammatory cascades, restoring metabolic energy homeostasis, and neutralizing oxidative stress, ultimately emerging as a highly viable candidate for targeted SCM intervention [5,6].

Cell death is a key component in the pathological process of sepsis and SCM and is mainly categorized into accidental cell death (ACD) and programmed cell death (PCD). PCD includes various types, such as apoptosis, necrosis, pyroptosis, ferroptosis, and cuproptosis, etc., and these different types of PCD play important roles in the pathogenesis of sepsis and SCM [7,8]. Specifically, emerging evidence underscores that the dysregulation of apoptosis, aberrant necroptosis, and impaired mitophagy profoundly exacerbate cardiac injury by amplifying localized inflammatory responses and disrupting mitochondrial homeostasis [9,10]. Furthermore, previous studies have indicated that Pue exerts significant cardioprotective effects across various disease models by directly targeting these PCD-related pathways—for instance, by alleviating apoptosis and modulating mitophagy to reduce inflammatory damage [11,12,13].

However, most of the current studies focus on exploring the role of a single type of PCD in SCM and lack a comprehensive analysis of multiple PCD mechanisms. Moreover, previous studies have indicated that Pue can play a therapeutic role in various diseases via targeting the PCD-related pathways, while the relationship between Pue and PCD in SCM remains unclear. The aim of this study is to explore the role of Pue in the regulation of multiple PCD mechanisms in SCM through a comprehensive analysis and to construct a diagnostic model based on it, so as to provide new ideas and methods for the early diagnosis and treatment of SCM.

## 2. Materials and Methods

### 2.1. Data Acquisition and Download

Within our analysis, eight cohorts of SCM and corresponding normal samples were downloaded from the public database, Gene Expression Omnibus [GEO, https://www.ncbi.nlm.nih.gov/geo/ (accessed on 15 May 2024)] [14], including GSE79962 (20 SCM vs. 11 normal), GSE9667 (6 SCM vs. 3 normal), GSE35934 (3 SCM vs. 3 normal), GSE40180 (5 SCM vs. 5 normal), GSE44363 (4 SCM vs. 4 normal), GSE53007 (4 SCM vs. 4 normal), GSE141864 (7 SCM vs. 3 normal), and GSE167363 (3 SCM vs. 2 normal). The detailed information of the above eight cohorts is exhibited in Table 1. Moreover, the selection criteria of genes related to PCD (PRGs) and targets of Pue are listed in the Supplementary Methods, and the comprehensive information of PRGs is exhibited in Table S1 [8,15,16,17,18,19,20,21,22].

### 2.2. Calculating the Weight of 14 PCD Patterns in SCM

Within the expression of 1515 PRGs in our training cohort, we conducted a single-sample gene set enrichment analysis (ssGSEA) via the R package “GSVA” (version 1.40.1) [23,24].

### 2.3. Transcriptomic Profiling and Statistical Thresholds

Evaluating the molecular divergence between normal specimens and those afflicted with SCM requires rigorous data stratification. For the GSE79962 cohort, we isolated critical transcriptomic shifts by establishing stringent significance boundaries. Biomarker candidates had to demonstrate a *p* under 0.05 alongside a minimum absolute log_2_ fold change (|log_2_FC|) of 1.0 to be officially classified as true differentially expressed genes (DEGs). Our analytical pipeline relied heavily on the R programming environment, deploying the limma package (release 3.56.2) for robust downstream processing [25]. We translated these high-dimensional statistical outputs into intuitive visual formats utilizing the ggplot2 library (version 3.5.2) [26].

### 2.4. Enrichment Analysis

To perform the enrichment analysis, we first retrieved the annotation of Gene Ontology (GO) and Kyoto Encyclopedia of Genes and Genomes (KEGG) database using the “org.Hs.eg.db” R package (version 3.17). Then, to complete the enrichment analysis for the candidate genes, the “clusterProfiler” R package (version 3.14.3) was used [27,28].

### 2.5. Construction of the Diagnostic Model via Machine Learning

To formulate a robust predictive framework, we constructed a diagnostic signature employing multivariate logistic regression. The individual risk quantification was calculated utilizing the following mathematical equation: Diagnostic Score = Σ_i_ (Coefficient_i_ × Expression level of feature_i_) [29]. Prior to finalizing this specific model, the previously isolated significant variables were evaluated across the clinical cohorts using an exhaustive computational strategy. This rigorous screening involved 13 independent machine learning techniques, which were systematically cross-paired to generate 171 distinct algorithmic pipelines. Comprehensive technical specifications regarding these comparative analyses are provided within the Supplementary Methods [30,31].

### 2.6. Immune Infiltration

Meanwhile, to investigate the infiltration level of the immune microenvironment, we collected the gene sets of 28 immune cells and 9 common immune-related pathways from the published studies and used the ssGSEA claimed above to calculate the infiltration score (Tables S2 and S3) [32,33].

### 2.7. Single Cell Analysis

To investigate how our key features are distributed across specific cell types, we retrieved the GSE167363 SCM dataset via the GEO. We subjected this single-cell cohort to a standard dimensionality reduction and clustering pipeline. Specifically, by executing algorithms for highly variable gene identification (FindVariableFeatures), data normalization (ScaleData), principal component analysis (PCA), and subsequent graph-based partitioning (FindNeighbors followed by FindClusters), the transcriptomic profiles were successfully stratified. Consequently, the analysis resolved ten distinct, fully annotated cellular populations. These identified groups encompassed neutrophils, macrophages, dendritic cells (DCs), megakaryocytes, monocytes, natural killer (NK) cells, B lymphocytes (B), CD8+ T cells (CD8 T), effector memory T (Tem) cells, alongside a generalized ‘Others’ classification [34].

### 2.8. Clinical Samples Collection

All research procedures adhered strictly to the ethical frameworks established by the Declaration of Helsinki. Prior to participant enrollment, the study protocol was formally authorized by the Ethics Committee at the Second Affiliated Hospital of Nanchang University, and written informed consent was successfully secured from all subjects. The locally recruited institutional cohort comprised 41 individuals in total: a control group of 20 healthy volunteers, alongside 21 critically ill patients diagnosed with SCM who were admitted to the intensive care unit (ICU). The included/excluded criteria for SMC patients were as follows: Adult patients meeting the Sepsis-3 diagnostic criteria, accompanied by newly diagnosed cardiac dysfunction (e.g., reduced ejection fraction or elevated cardiac biomarkers) not attributable to other causes were included in our study while patients with a prior history of coronary artery disease, chronic heart failure, primary cardiomyopathies, severe renal/hepatic failure, or active malignancies were excluded. From this population, we acquired peripheral blood specimens and their matched clinical records for subsequent downstream evaluation.

### 2.9. Animal Experiments

The study subjects consisted of three-week-old C57BL/6 mice, with an average body weight of 10 ± 1.5 g, which were acquired from the Model Animal Research Center at Nanjing University in China. Following initial acclimatization, the animals were subjected to randomized allocation to form two distinct experimental arms: a baseline control cohort (NC, n = 6) and a disease induction group (SCM, n = 8). To effectively establish the pathological model, subjects in the SCM arm received an intraperitoneal administration of lipopolysaccharide (LPS; Sigma) at a targeted dosage of 10 mg/kg. Upon completion of the required observational period, humane euthanasia was performed to facilitate downstream tissue collection; specifically, the animals were heavily sedated utilizing a 3% isoflurane gas mixture prior to physical termination via cervical dislocation.

### 2.10. Echocardiographic Assessment

Cardiac performance was evaluated using a Vevo 2100 high-resolution ultrasound system equipped with an MS-550D transducer (VisualSonics, Toronto, ON, Canada). Initial structural assessments of the murine hearts were captured via B-mode (two-dimensional) acquisitions oriented along the parasternal short-axis. Subsequently, M-mode tracings were recorded to quantify key functional metrics. This approach allowed for the determination of left ventricular internal diameters during both the systolic (LVDs) and diastolic (LVDd) phases, facilitating the calculation of fractional shortening (FS%) and ejection fraction (EF%). Throughout the duration of the non-invasive imaging procedures, continuous sedation was maintained utilizing a gas mixture containing 2% isoflurane and oxygen.

### 2.11. Hematoxylin-Eosin (HE) Staining

To quantify the morphological cytoplasm-to-nucleus ratio, digital histological evaluations were conducted utilizing ImageJ software (version 1.54p 17). This computational analysis specifically delineated the nuclear compartments (stained purple) from the surrounding cytoplasmic domains (stained red). Quantitative assessments were performed on representative micrographs, which were acquired at magnifications of 20× and 40× from anatomically consistent regions across the myocardial samples. Prior to the optical imaging, the cardiac specimens underwent a standardized preparation pipeline. Initially, the heart tissues were preserved utilizing a 4% paraformaldehyde fixative, followed by routine processing for paraffin embedding and subsequent microtome sectioning at a 5-μm thickness. Finally, these slides were processed using a commercial HE assay (Solarbio, Beijing, China), with all procedural steps executed in strict adherence to the manufacturer’s supplied protocol.

### 2.12. Reverse Transcription Quantitative Polymerase Chain Reaction (RT-qPCR)

The method for RT-qPCR was described in our previously published paper [35]. Meanwhile, the primer pairs utilized in our article were listed in Table S4.

### 2.13. Statistical Analysis

When comparing continuous data across two independent cohorts, the unpaired Student’s *t*-test was utilized to determine statistical variance. All computational processing, data management, and graphical visualizations were executed employing R software (version 4.3.1) in conjunction with GraphPad Prism (version 9.5). Across all analytical procedures within this study, a two-sided P of less than 0.05 was established as the criterion for statistical significance.

## 3. Results

### 3.1. General Landscape of PCD in SCM

**Figure 1:**
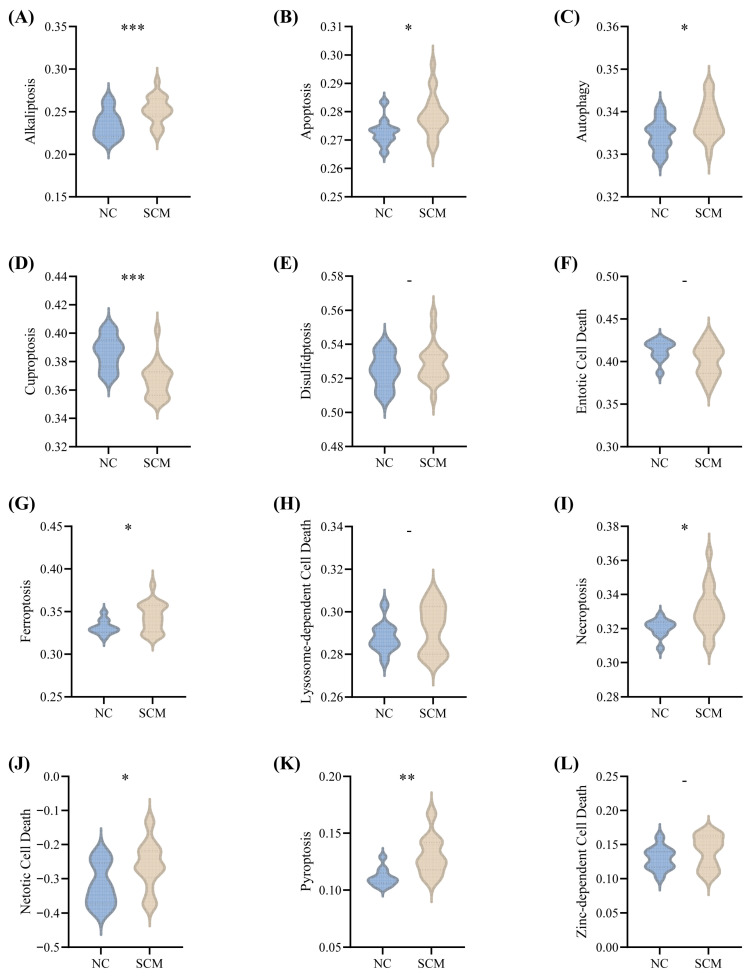
General landscape of PCD in the training cohort, GSE79962. The panels illustrate the differential ssGSEA weights between the normal control (NC) and SCM groups for specific PCD patterns, including: (**A**) Alkaliptosis; (**B**) Apoptosis; (**C**) Autophagy; (**D**) Cuproptosis; (**E**) Disulfidptosis; (**F**) Entotic cell death; (**G**) Ferroptosis; (**H**) Lysosome-dependent cell death; (**I**) Necroptosis; (**J**) Netotic cell death; (**K**) Pyroptosis; and (**L**) Zinc-dependent cell death. -, *p* ≥ 0.05; *, *p* < 0.05; **. *p* < 0.01; ***. *p* < 0.001.

Utilizing a comprehensive panel of 1515 genes associated with PCD, we evaluated the baseline activation status of 14 distinct PCD modalities within the GSE79962 training cohort. Because two of these functional categories yielded undetectable activity levels, they were excluded from subsequent analytical steps. For the remaining 12 modalities, comparative profiling was conducted to delineate the transcriptomic divergence between the NC and SCM groups. Our quantitative assessments revealed that nine of these specific patterns experienced statistically significant alterations (*p* < 0.05; Figure 1A–L). Ultimately, this widespread immunological dysregulation strongly underscores the fundamental pathophysiological contribution of PCD mechanisms to the progression of SCM.

### 3.2. Obtaining the PCD-Related Targets of Puerarin in SCM

**Figure 2:**
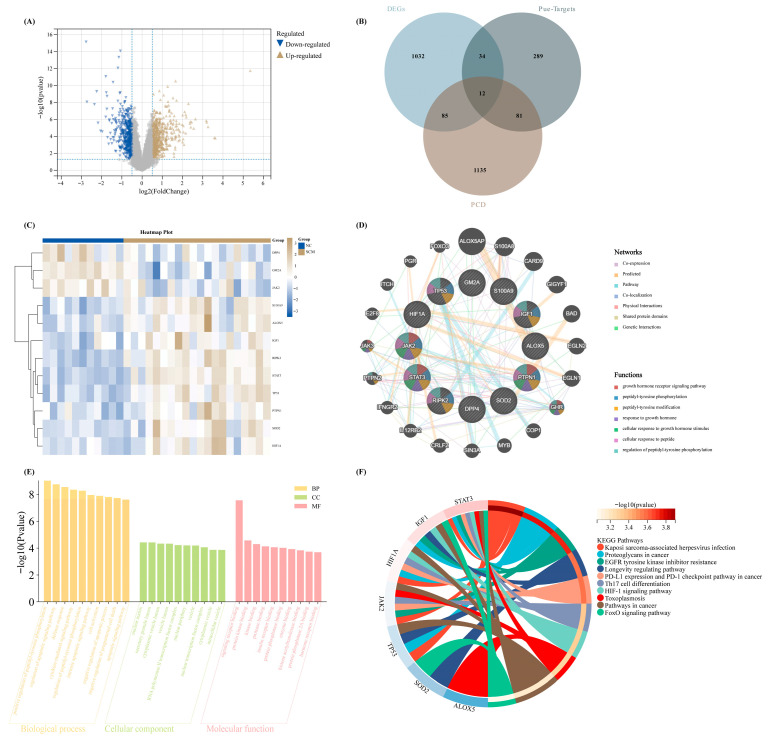
Initial identification and functional characterization of Pue-modulated, PCD-related biomarkers in septic cardiomyopathy. (**A**) Transcriptomic variations distinguishing NC from SCM samples within the GSE79962 discovery dataset are visually depicted via a volcano plot. Grey simbols mean genes without significant expression difference. (**B**) By cross-referencing these DEGs with established PRGs and predicted pharmacological targets of Pue, an intersection analysis highlights exactly twelve core candidate features. (**C**) The relative transcriptional abundance of this specific 12-gene signature across the cohort is detailed in a hierarchical heatmap. (**D**) Meanwhile, the underlying biological crosstalk and molecular interactions among these specific targets are mapped out using a co-expression network model. To further elucidate the broader mechanistic roles of these isolated features, comprehensive functional annotations were performed, with the resulting biological processes and signaling cascades summarized through GO (**E**) and KEGG (**F**) enrichment diagrams.

According to the key therapeutic role of Pue and the crucial characteristic of PCD in SCM, we presumed that Pue can play an important role in the treatment of SCM via regulating PCD. Therefore, we aimed to obtain the PCD-related targets of Pue in SCM. Firstly, the DEGs between SCM and NC in our training cohort were identified (Figure 2A). Furthermore, via intersecting the targets of Pue and the genes of the above 9 PCD patterns, a total of 12 PCD-related targets of Pue were obtained (Figure 2B). The expression heatmap in GSE79962 of these 12 elements is shown in Figure 2C. Meanwhile, we constructed a network to reveal the interaction relationship among these 12 targets (Figure 2D). Moreover, we performed the GO and KEGG enrichment analysis for the 12 elements, in which pathways related to immune, inflammation, and cell death were the major events (Figure 2E,F).

### 3.3. Identification of Crucial Targets and Construction of the Diagnostic Model for SCM

**Figure 3:**
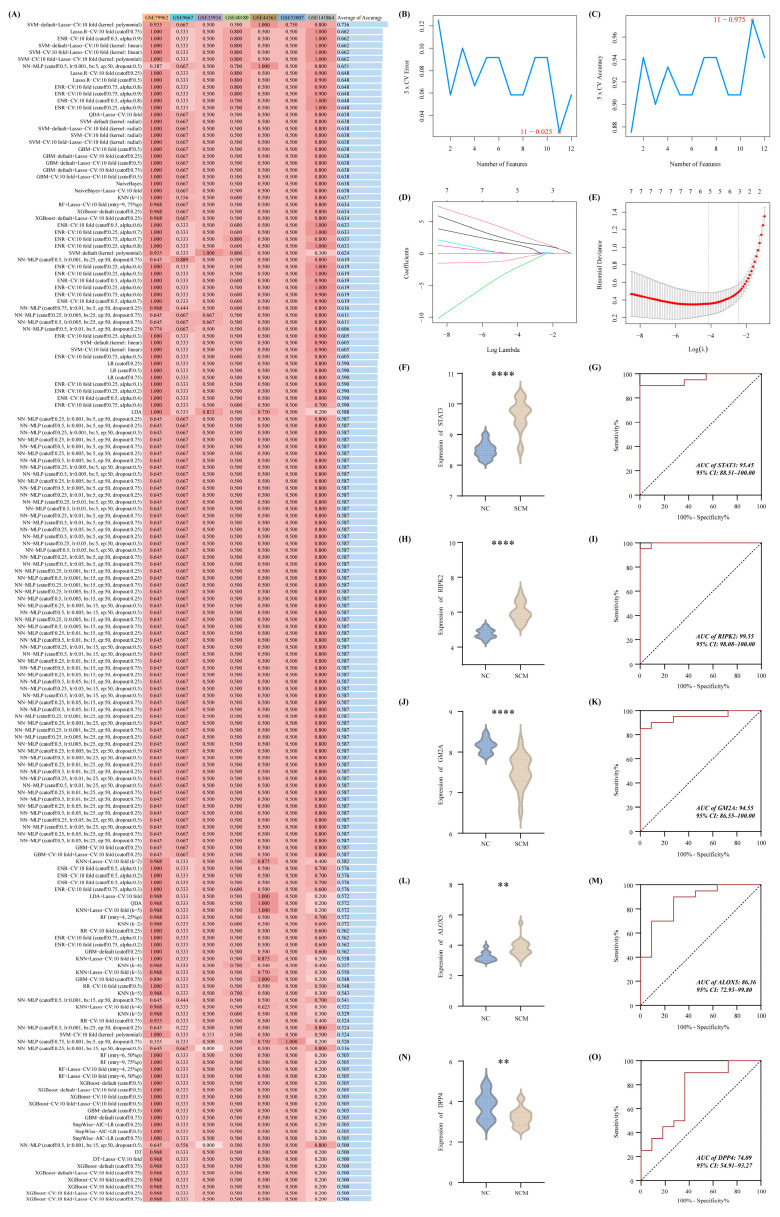
Machine learning-driven isolation of core Puerarin-associated, programmed cell death-related biomarkers in septic cardiomyopathy. (**A**) A comprehensive evaluation of 171 distinct algorithmic combinations, derived from 13 independent computational methodologies, was conducted to pinpoint key predictive features and determine their overall predictive accuracy across the analyzed cohorts. (**B**,**C**) Performance metrics for the SVM-RFE approach, detailing the corresponding error rate trajectory (**B**) alongside the accuracy profile that ultimately facilitated the extraction of an 11-gene subset (**C**). (**D**,**E**) Subsequent refinement utilizing the Least Absolute Shrinkage and Selection Operator (LASSO) regression. Panel (**D**) illustrates the dynamic coefficient trajectories of the remaining candidate transcripts, while panel (**E**) maps the partial likelihood deviance used to finalize the optimal five-feature diagnostic signature. (**F**–**O**) Independent validation of the five prioritized biomarkers within the primary GSE79962 discovery dataset. For each specific target—STAT3 (**F**,**G**), RIPK2 (**H**,**I**), GM2A (**J**,**K**), ALOX5 (**L**,**M**), and DPP4 (**N**,**O**)—the panels sequentially display their relative transcriptional disparities between normal controls and diseased subjects, immediately followed by their respective ROC curves to assess individual discriminatory capacity. **. *p* < 0.01; ****, *p* < 0.0001.

To pinpoint the most robust biomarkers for SCM from our initial pool of twelve candidates, a comprehensive computational screening strategy was deployed. Transcriptomic profiles from this dozen-feature set served as the foundational input matrix. We evaluated 171 unique algorithmic pipelines—generated by systematically cross-pairing 13 distinct machine learning methodologies. These models were trained on the primary discovery cohort (GSE79962) and subsequently tested across six independent external validation arrays (GSE35934, GSE9667, GSE44363, GSE40180, GSE141864, alongside GSE53007). Performance benchmarking established that a hybrid modeling pipeline, specifically integrating a default SVM with a 10-fold cross-validated LASSO regression employing a polynomial kernel, achieved the highest mean accuracy score of 0.736 (Figure 3A). Utilizing this optimized framework, the preliminary SVM filtering stage successfully distilled the candidate pool down to eleven relevant transcripts (Figure 3B,C). Subsequent application of the LASSO algorithm refined this subset even further, ultimately isolating five highly critical diagnostic determinants: ALOX5, DPP4, GM2A, RIPK2, and STAT3 (Figure 3D,E). The differential abundance profiles and ROC evaluations for this final pentagene signature are fully detailed in Figure 3F–O.

**Figure 4:**
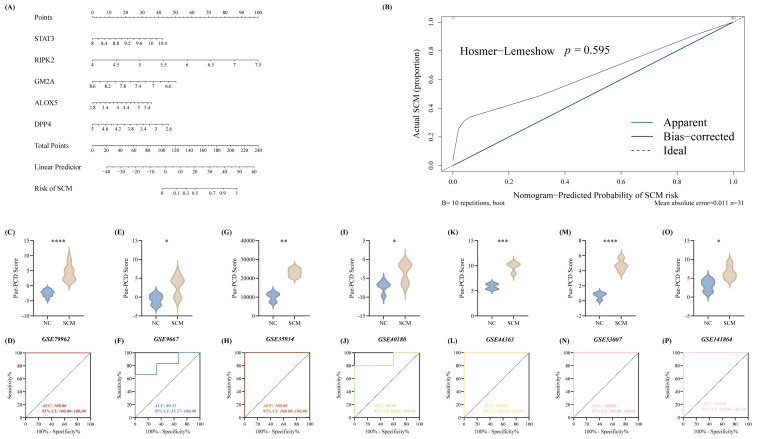
Development and independent verification of the Pue-associated PCD predictive signature for SCM. (**A**) A visual risk-assessment nomogram incorporating the five core biomarkers (ALOX5, DPP4, GM2A, RIPK2, and STAT3) to quantitatively estimate disease probability. (**B**) Calibration plotting used to assess the agreement between the nomogram’s predicted risks and the actual observed clinical outcomes. (**C**,**D**) Baseline performance metrics within the primary GSE79962 discovery dataset, illustrating the statistical variance in calculated risk scores between NC and SCM subjects (**C**), alongside the corresponding ROC profile demonstrating initial diagnostic efficacy (**D**). (**E**–**P**) Extensive external validation of the derived predictive framework across six independent cohorts (GSE9667, GSE35934, GSE40180, GSE44363, GSE53007, and GSE141864). To confirm the model’s robust discriminatory capacity across diverse clinical populations, the comparative distribution of patient diagnostic scores is presented for each respective dataset (**E**,**G**,**I**,**K**,**M**,**O**), immediately followed by their associated ROC analyses (**F**,**H**,**J**,**L**,**N**,**P**). *, *p* < 0.05; **. *p* < 0.01; ***. *p* < 0.001; ****, *p* < 0.0001.

To formulate a robust Pue-mediated PCD-related risk signature for distinguishing SCM from normal controls (NC), candidate features were subjected to logistic regression analysis. This computational approach yielded a quantitative diagnostic tool, visually represented as a nomogram (Figure 4A), which is driven by the following algorithm: Diagnostic Score = (Expression_STAT3_ × 1.4595327) + (Expression_RIPK2_ × 2.1563116) − (Expression_GM2A_ × 2.9607514) + (Expression_ALOX5_ × 0.4433711) − (Expression_DPP4_ × 0.6460931). Assessment of this predictive framework within the GSE79962 demonstrated exceptional discriminatory capacity. Application of the derived formula revealed significantly elevated risk scores in the SCM patient population compared to NC (*p* < 0.0001; Figure 4C). Furthermore, the area under the curve (AUC) reached a flawless 100.00 in this discovery cohort (Figure 4D). Favorable agreement between predicted probabilities and actual clinical outcomes was also confirmed via calibration plotting, which showed no significant deviation from the ideal reference line (*p* = 0.595; Figure 4B).

To rigorously verify the generalizability of this multigene signature, we subsequently evaluated its performance across six independent external validation sets. Consistently, the model successfully discriminated SCM subjects from NC individuals with high statistical confidence across all supplementary cohorts (Figure 4E,G,I,K,M,O). This strong diagnostic utility was further corroborated by the corresponding ROC analyses, yielding robust AUC values of 86.33 for GSE9667, 88.00 for GSE40180, and perfect 100.00 scores for GSE35934, GSE44363, GSE53007, and GSE141864 (Figure 4F,H,J,L,N,P).

### 3.4. The Investigation of the Immune Landscape in SCM

**Figure 5:**
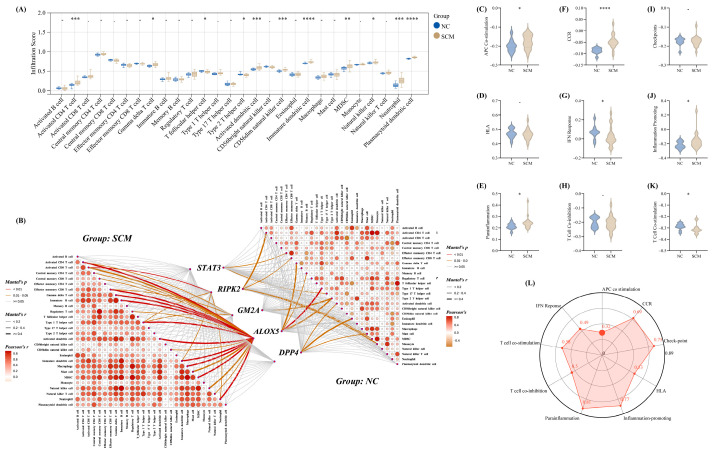
Immunological microenvironment profiles and their modulation by Pue and PCD within the SCM context. (**A**) Relative abundance levels for 28 distinct leukocyte subpopulations were computed utilizing single-sample gene set enrichment analysis (ssGSEA), highlighting variations between healthy controls (NC) and disease states within the GSE79962 discovery dataset. (**B**) A heatmap derived from Mantel tests visualizes how the transcriptomic profiles of the five critical Pue-associated PCD targets correlate with broader immune infiltration patterns. (**C**–**K**) Comparative assessments detailing functional activation disparities across normal and pathological groups for nine specific immunological cascades: APC co-stimulation (**C**), CC chemokine receptor (CCR) signaling (**D**), immune checkpoints (**E**), human leukocyte antigen (HLA) complex (**F**), inflammation promotion (**G**), parainflammation (**H**), T-lymphocyte co-inhibition (**I**), T-lymphocyte co-stimulation (**J**), and interferon (IFN) responsiveness (**K**). (**L**) A multidimensional radar plot mapping the statistical dependencies linking the overall diagnostic signature to the activation statuses of the aforementioned nine immune cascades. *, *p* < 0.05; **. *p* < 0.01; ***. *p* < 0.001; ****, *p* < 0.0001.

Initial profiling of the training cohort highlighted a stark contrast in the immunological microenvironments of the SCM and NC groups. Specifically, mapping the infiltration patterns of distinct immune cell populations revealed a robustly stimulated state within the SCM samples (Figure 5A). This heightened immunological activity was further corroborated by an assessment of nine canonical immune pathways, all of which demonstrated significant upregulation in the disease cohort compared to the healthy controls (Figure 5C,K). Moreover, to elucidate the regulatory connection between our identified biomarkers and this localized inflammation, we evaluated the interactions involving the five key Pue-mediated, PCD-associated genes. Mantel tests indicated that the transcriptional profiles of these five targets shared a substantially stronger association with the overarching immune infiltration scores in the SCM condition than in the normal state (Figure 5B). Consistently, subsequent evaluations uncovered a strong positive linear relationship linking the integrated diagnostic score to the activation levels of the aforementioned nine immune pathways (Figure 5L). Together, these findings underscore a pivotal mechanistic link between the diagnostic gene signature and the exacerbated immune responses characteristic of SCM.

**Figure 6:**
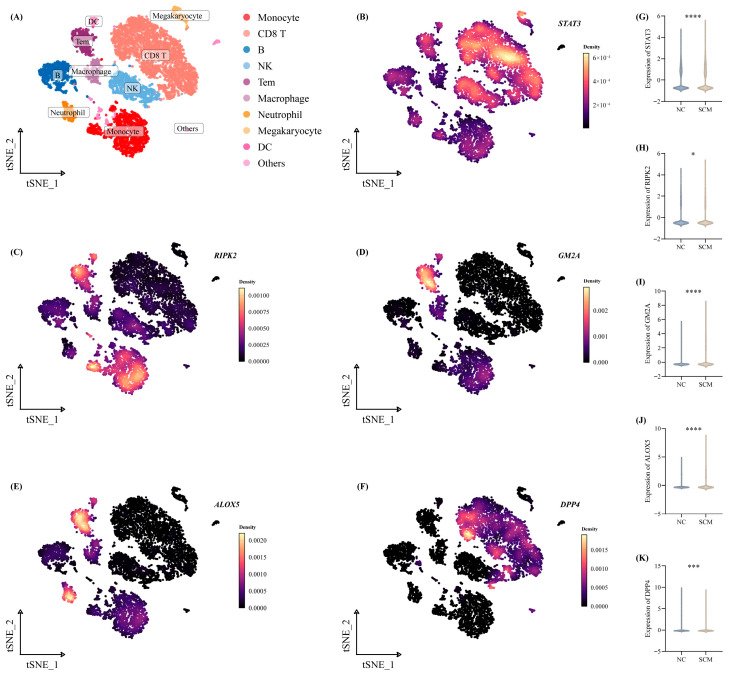
The expression status of the five significant Pue-PCD features in the single-cell dataset. (**A**) The annotated tSNE plot visualizing different cell clusters in the single-cell dataset. (**B**) The expression density of STAT3 across cell clusters. (**C**) The expression density of RIPK2 across cell clusters. (**D**) The expression density of GM2A across cell clusters. (**E**) The expression density of ALOX5 across cell clusters. (**F**) The expression density of DPP4 across cell clusters. (**G**) Violin plot showing the detailed expression of STAT3 in each cell type for NC vs. SCM. (**H**) Violin plot showing the detailed expression of RIPK2 in each cell type. (**I**) Violin plot showing the detailed expression of GM2A in each cell type. (**J**) Violin plot showing the detailed expression of ALOX5 in each cell type. (**K**) Violin plot showing the detailed expression of DPP4 in each cell type. *, *p* < 0.05; ***. *p* < 0.001; ****, *p* < 0.0001.

The overarching distribution of ten distinct cellular populations—encompassing macrophages, DC, NK cells, B, CD8 T, monocytes, megakaryocytes, and other cells—across the analyzed specimens is visualized in Figure 6A. We leveraged this specific single-cell cohort to further elucidate the spatial and cell-type-specific transcriptional profiles of our core gene signature within the SCM context. Notably, comparative evaluations between the SCM and NC groups demonstrated that the five key biomarkers (STAT3, RIPK2, GM2A, ALOX5, and DPP4) exhibited highly variable, population-dependent expression patterns (Figure 6B–K). These heterogeneous localizations strongly suggest that these candidate targets exert multifaceted and pivotal regulatory effects on the localized immunological niche.

### 3.5. Experimental Verification of the Core Pue-PCD Signature and Its Association with Cardiac Performance

**Figure 7:**
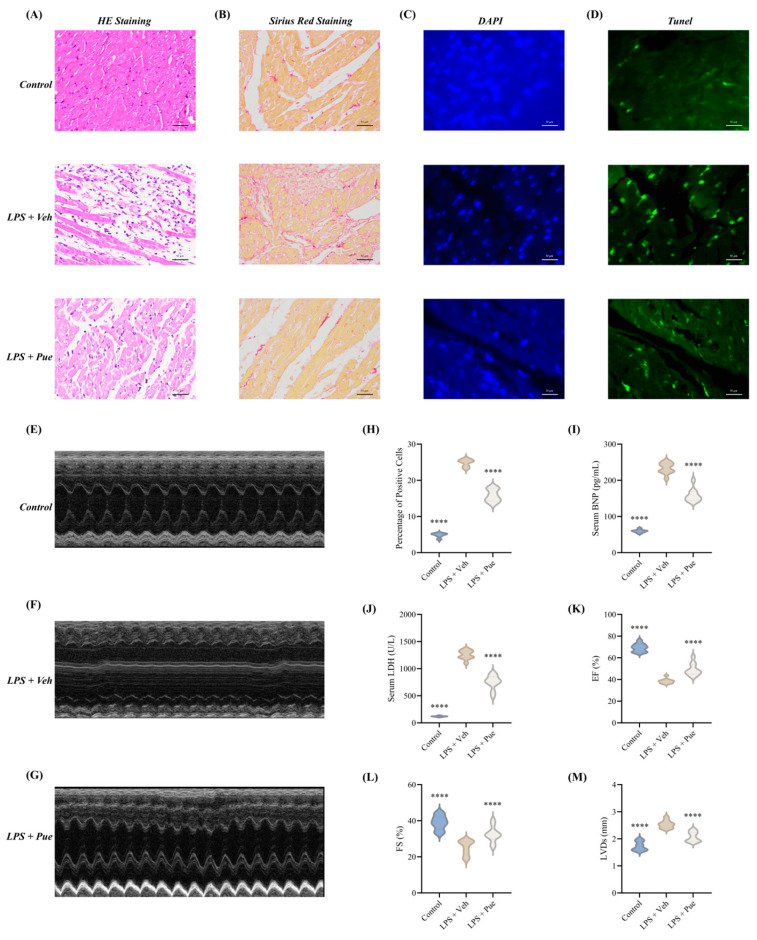
The therapeutic role of Pue in the SCM animal model. (**A**) Representative HE staining images of heart tissue. (**B**) Sirius Red staining indicates myocardial fibrosis. (**C**) DAPI staining. (**D**) TUNEL staining showing cardiomyocyte apoptosis (green fluorescence). (**E**–**G**) Representative M-mode echocardiographic tracings for (**E**) Control, (**F**) LPS model, and (**G**) LPS mice model with Pue. (**H**) Quantification of TUNEL-positive cells (n = 3 per group, ANOVA test). (**I**) Serum BNP levels (n = 6–8, ANOVA test). (**J**) Serum LDH levels (n = 6–8, ANOVA test). (**K**–**M**) Echocardiographic assessment of cardiac function: (**J**) EF%, (**K**) FS%, and (**L**) LVDs (n = 6–8 per group, ANOVA test). ****. *p* < 0.0001.

To rigorously evaluate the biological relevance of the identified gene signature, an in vivo lipopolysaccharide (LPS)-induced murine model of SCM was initially established. Successful disease induction was phenotypically verified by the pronounced observation of myocardial damage and localized inflammatory responses within the experimental cohort. Moreover, with the usage of Pue, the above abnormal injury of the myocardium was alleviated, emphasizing the protective role of Pue in SCM (Figure 7).

**Figure 8:**
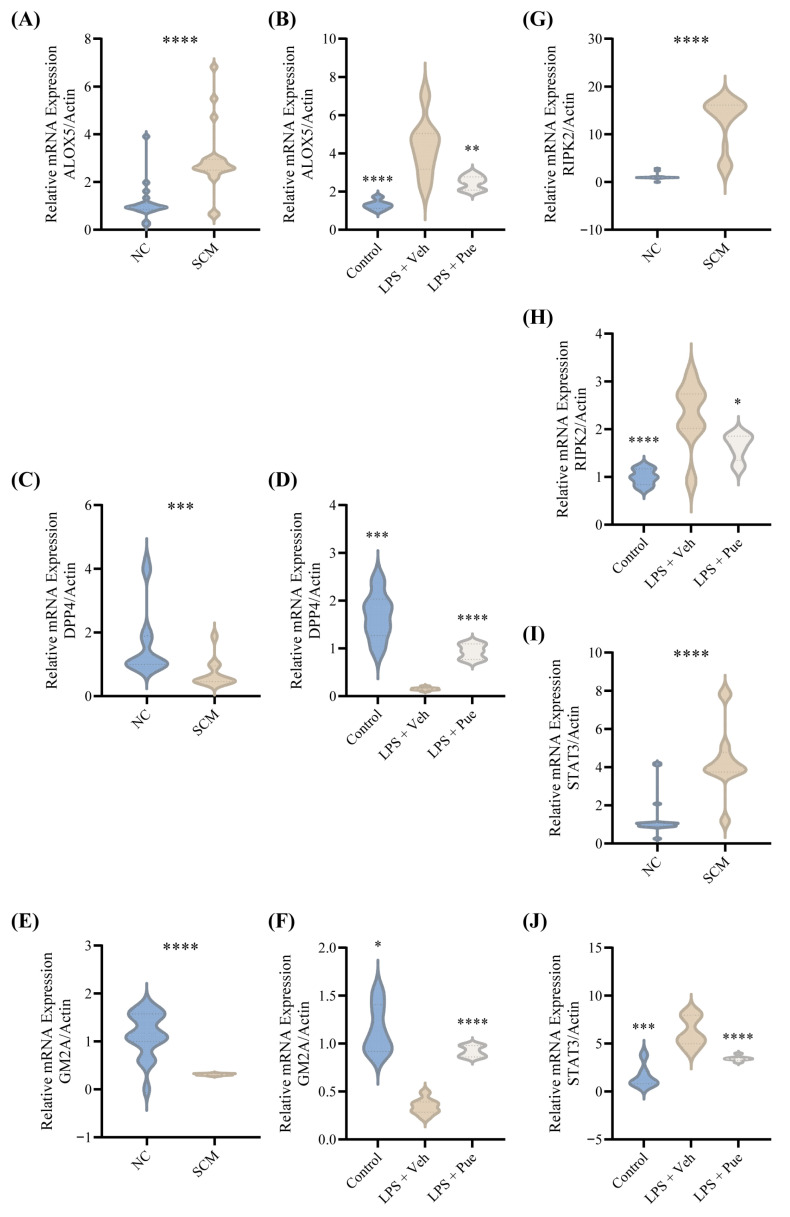
Validation of the five crucial Pue-PCD features in both human and animal models. The relative mRNA expression levels of (**A**,**B**) ALOX5, (**C**,**D**) DPP4, (**E**,**F**) GM2A, (**G**,**H**) RIPK2, and (**I**,**J**) STAT3 in (**A**,**C**,**E**,**G**,**I**) peripheral blood samples from SCM patients (n = 21) compared to healthy controls (n = 20) and in (**B**,**D**,**F**,**H**,**J**) heart tissues of SCM mice (n = 8) compared to the Control group (n = 6), analyzed via RT-qPCR. Data are presented as violin plots. Statistical significance was determined using an unpaired *t*-test and ANOVA test. *, *p* < 0.05; **. *p* < 0.01; ***. *p* < 0.001; ****, *p* < 0.0001.

Clinical translatability was assessed by measuring these identical elements within peripheral blood samples collected from human SCM patients. Crucially, the RT-qPCR expression profiles derived from this clinical cohort (Figure 8A,C,E,G,I) mirrored the patterns observed in the solid analysis. Following this pathological confirmation, ventricular tissues were harvested from the subjects to quantify the transcriptional abundance of the five candidate biomarkers via RT-qPCR. These assays demonstrated highly significant differential expression between the LPS-treated animals and the healthy controls across all five targets. And Pue also resisted the abnormal expression of the above five biomarkers, revealing the potential regulation relationship between Pue and these five elements (Figure 8B,D,F,H,J). Collectively, both the human systemic data and the localized animal experimental datasets perfectly corroborated the directional trends initially predicted by our computational bioinformatics framework.

Furthermore, to evaluate the physiological impact of the five selected Pue-PCD biomarkers on cardiac hemodynamics, we conducted associative analyses comparing their localized transcriptional profiles against six functional parameters derived from the experimental SCM cohort. As detailed within Table 2, the expression levels for every single candidate gene demonstrated statistically significant correlations with the measured myocardial indices. These robust associations firmly substantiate the essential regulatory involvement of these specific targets in modulating ventricular performance during disease progression.

## 4. Discussion

In this study, we found that nine PCD modalities play an important role in the onset and progression of SCM. Notably, existing studies have clearly indicated that Apoptosis, Autophagy, Ferroptosis, Necroptosis, and Pyroptosis, the five PCD modalities, have a significant driving role in the pathologic process of SCM, which is highly consistent with our findings [36,37,38,39]. Meanwhile, we integrated PCD gene sets and Pue targets based on the disease characteristics of SCM, screened the genes and performed functional and pathway exploration, and there was an enrichment mainly in the features of immune, inflammation, and cell death, which suggests that Pue modulates PCD patterns in SCM, which is closely related to inflammatory response. Subsequently, we combined multiple machine learning algorithms to further screen 5 crucial features, *STAT3*, *RIPK2*, *GM2A*, *ALOX5* and *DPP4*, of which the specific mechanism of action of GM2A has not been reported in SCM.

Recent evidence highlights the therapeutic promise of suppressing the *JAK2/STAT3* cascade, as this targeted intervention substantially mitigates inflammatory damage in cardiomyocytes exposed to LPS [40]. Conversely, when this signaling axis is actively upregulated, it accelerates the clinical deterioration associated with SCM. This disease progression is driven by a cascade of detrimental cellular events, specifically the induction of mitochondrial impairment, heightened oxidative stress, accelerated cellular senescence, and a magnified localized inflammatory state [41]. The profound impact of this pathway on cardiac pathology stems from the fundamental biological roles of its core component. The *STAT3* gene produces a crucial intracellular mediator that relies on *JAK* to modulate downstream transcriptional activity. Under normal physiological conditions, this protein orchestrates a vast array of essential functions, encompassing metabolic control, immunological responses, and the regulation of cellular lifespans—including proliferation and differentiation. Consequently, maintaining the precise equilibrium of *STAT3* is imperative for overall systemic well-being, as both its anomalous silencing and excessive stimulation are intimately linked to various pathological conditions [42].

*RIPK2* encodes receptor-interacting protein kinase-2, a key signaling protein downstream of *NOD1/2* involved in the recognition and transduction of bacterial infection signals. After recognizing conserved features of the bacterial cell wall, *NOD1* and *NOD2* activate *RIP2* through its CARD structural domain, which in turn initiates the inflammatory signaling pathway [43]. It has been shown that *RIP2* also induces inflammatory responses by regulating the *TAK1/IκBα/NF-κB* signaling pathway [44]. Pro-inflammatory cytokines (e.g., *TNF-α*, *IL-1α*, *IL-1β*, and *IL-6*) are overproduced in the induced inflammatory response, which can impair cardiomyocyte function and lead to decreased myocardial contractility. And *RIP2* knockdown or inhibition can attenuate LPS-induced cardiac dysfunction.

*ALOX5* encodes 5-lipoxygenase (*5-Lox*), which catalyzes the generation of leukotriene B4 (*LTB4*) from arachidonic acid (AA), activates the *BLT1/IL-12 p35* pathway, increases the production of inflammatory factors, and promotes inflammatory responses. It has been shown that *ALOX5* plays a central role in PCD, including apoptosis, pyrophosphorylation and ferroptosis [45]. Activation of 5-Lox promotes the activation of cardiomyocyte apoptotic markers (e.g., caspase-3), leading to increased cardiomyocyte apoptosis, as well as enhanced polarization of M1-type macrophages [46]. Inhibition of *5-Lox*, either by pharmacological inhibition or gene knockout, significantly ameliorated sepsis-induced cardiac dysfunction and reduced myocardial injury.

*DPP4* encodes dipeptidyl peptidase-4, which degrades a large number of cytokines, chemokines, hormones, and growth factors, and plays a role in T cell activation and proliferation. It has been found that a significant decrease in serum *DPP4* activity occurs in sepsis patients compared to controls across a series of time gradients and that there is a significant correlation between *DPP4* activity and sepsis survival, with higher *DPP4* activity being associated with higher survival rates [47]. Thus, we hypothesized that *DPP4* may have a protective effect against septic cardiomyopathy, although some studies have held the opposite opinion, suggesting that inhibition of *DPP4* reduces ROS production, preserves mitochondrial membrane potential, and restores mitochondrial bioenergetic function, attenuates oxidative stress damage to cardiomyocytes, and has a protective effect against cardiovascular disease [48,49]. Its specific mechanism in SCM needs further research.

To facilitate early clinical detection, the quintet of identified biomarkers was leveraged to formulate a robust predictive risk framework. This quantitative signature displayed exceptional discriminatory capacity not only within the primary discovery cohort but also across six independent replication datasets, offering highly promising translational avenues. Mechanistically, functional annotations of the overlapping gene profiles imply that Pue modulates PCD dynamics during SCM progression primarily by regulating severe inflammatory cascades. Subsequent evaluations of the overarching immunological landscape confirmed an exacerbated inflammatory state within the pathological tissues. Furthermore, statistical assessments uncovered a strong positive association linking the calculated diagnostic scores directly with the activation of localized host defense pathways, reinforcing the premise that localized inflammation serves as a fundamental driver of Pue-mediated protective mechanisms. To resolve these biological interactions at a higher resolution, single-cell transcriptomic analyses were integrated. This approach revealed a highly heterogeneous distribution of the core pentagene signature across various leukocyte subsets, indicating that these specific molecular targets exert distinct, multifaceted regulatory effects within the surrounding immune niche. Finally, to rigorously verify these computational findings in vivo, an experimental murine model of septic cardiomyopathy was established, whereby the transcriptional alterations of all five candidate markers were successfully validated utilizing RT-qPCR.

However, there were also several limitations in this study. First, while we identified *STAT3*, *RIPK2*, *GM2A*, *ALOX5*, and *DPP4* as core targets associated with the protective effects of Pueraria in SCM, our findings are based on transcriptomic changes and expression validation. And the lack of a “non-SCM ICU sepsis patient” means we cannot definitively conclude that these gene alterations are completely specific to SCM rather than general severe infection. Second, regarding the machine learning construction, the sample size of the SCM cohorts available in public databases is relatively small, which may introduce potential bias or overfitting. Although we utilized multiple validation sets to mitigate this, the generalization of the model requires further verification in larger-scale clinical cohorts. Finally, the cross-species validation (mouse models and human datasets) provides strong support, but species-specific immune differences and the validation or functional experiments at the protein level should be considered when translating these findings to clinical applications.

## 5. Conclusions

In our study, the cellular and molecular mechanisms of Pue in regulating PCD patterns in SCM were explored. The identification and validation of *STAT3*, *RIPK2*, *GM2A*, *ALOX5*, and *DPP4* as potential molecular targets provide evidence for further studies on early diagnosis and treatment of SCM. Meanwhile, we also found that Pue was closely associated with inflammatory response in modulating PCD patterns in SCM. This has potential therapeutic value that needs to be explored in further studies.

## Figures and Tables

**Table 1:** The basic information about the data sets utilized in our research.

Dataset ID	Platform	Type	Sample Size		Species	Tissue
			Normal	SCM		
GSE79962	GPL6244	Training	11	20	Human	Heart
GSE9667	GPL339	Validation	3	6	Mouse	Heart
GSE35934	GPL6845	Validation	3	3	Mouse	Heart
GSE40180	GPL6887	Validation	5	5	Mouse	Heart
GSE44363	GPL1261	Validation	4	4	Mouse	Heart
GSE53007	GPL6885	Validation	4	4	Mouse	Heart
GSE141864	GPL17586	Validation	3	7	Human	Heart
GSE167363	GPL24676	SC-seq Set	2	3	Human	Blood

**Table 2:** Statistical dependencies linking core biomarker expression profiles with established physiological indices in the SCM model.

	BNP		LDH		EF		FS		LVDs		LVDd	
	Coe	*p*	Coe	*p*	Coe	*p*	Coe	*p*	Coe	*p*	Coe	*p*
*ALOX5*	0.802	0.001	0.807	<0.001	−0.809	<0.001	−0.753	0.002	0.727	0.003	−0.039	0.896
*STAT3*	0.898	<0.001	0.894	<0.001	−0.838	<0.001	−0.843	<0.001	0.876	<0.001	0.368	0.195
*RIPK2*	0.829	<0.001	0.825	<0.001	−0.771	0.001	−0.677	0.008	0.793	0.001	0.449	0.108
*GM2A*	−0.918	<0.001	−0.920	<0.001	0.914	<0.001	0.809	<0.001	−0.895	<0.001	−0.029	0.921
*DPP4*	−0.933	<0.001	−0.930	<0.001	0.934	<0.001	0.917	<0.001	−0.838	<0.001	−0.226	0.358

## Data Availability

The datasets (GSE79962, GSE9667, GSE35934, GSE40180, GSE44363, GSE53007, GSE141864, and GSE167363) analyzed during the current study are available in the repository of the Gene Expression Omnibus [https://www.ncbi.nlm.nih.gov/geo/ (accessed on 15 May 2024)].

## Supplementary Materials

The following supporting information can be downloaded at: https://www.mdpi.com/article/10.3390/biomedicines14051114/s1, Table S1: The introduction of 1515 genes related to each PCD type; Table S2: The gene sets of 28 immune cells used for ssGSEA; Table S3: The gene sets of 9 immune-related pathways used for ssGSEA; Table S4: The primer pairs utilized in Real-Time Quantitative PCR; Supplementary Methods: The detailed information for genes acquisition and machine learning.

## Abbreviations

The following abbreviations are used in this manuscript:

MODS	Multiple organ dysfunction syndrome
SCM	Septic cardiomyopathy
ACD	Accidental cell death
PCD	Programmed cell death
GEO	Gene Expression Omnibus
PRGs	Genes related to PCD
ssGSEA	Single-sample gene set enrichment analysis
DEGs	Differentially expressed genes
|log_2_FC|	Absolute log_2_ fold change
GO	Gene ontology
KEGG	Kyoto Encyclopedia of Genes and Genomes
PCA	Principal component analysis
CD8 T	CD8+ T cells
B	B cells
NK	Natural killer cells
Tem	Effect memory T cells
DC	Dendritic cells
ICU	Intensive care unit
LVDd	LV dimensions in diastole
LVDs	LV dimensions in systole
EF%	Percentage ejection fraction
FS%	Percentage fractional shortening
HE	Hematoxylin eosin
RT-pPCR	Reverse transcription quantitative polymerase chain reaction
NC	Normal control
5-LOX	5-lipoxygenase
LBT4	Leukotriene B4
AA	Arachidonic acid
