# Supplementary material for: Uncovering the Targets of Pueraria Associated with Programmed Cell Death and the Construction of a Diagnostic Model in Septic Cardiomyopathy
Source: Biomedicines. 2026 May 14;14(5):1114. doi: 10.3390/biomedicines14051114 (PMC13205065; doi:10.3390/biomedicines14051114)
Supplement: Supplementary file 1 [file biomedicines-14-01114-s001.zip › Supplementary Method.pdf]

Genes Acquisition

Within the key word (*programmed cell death*), we searched genes related to PCD (PRGs) in various databases and previous literature, including Molecular Signatures Database (MSigDB), GeneCards, Kyoto Encyclopedia of Genes and Genomes (KEGG), and review articles. Finally, a total of 1515 PRGs participated in 14 different kinds of PCD patterns were included in our analysis: 580 genes of apoptosis, 52 of pyroptosis, 88 of ferroptosis, 368 of autophagy, 101 of necroptosis, 14 of cuproptosis, 24 of disulfidptosis, 9 of parthanatos, 15 of entotic cell death, 8 of netotic cell death, 220 of lysosome-dependent cell death, 7 of alkaliptosis, 6 of oxeiptosis, 25 of zinc-dependent cell death.

The target of Pue was searched in TCMSP (https://old.tcmsp-e.com/tcmsp.php/) using "puerarin" as the key word. Canonical SMILES and chemical structural formulae of Pue were obtained in Pubchem (https://pubchem.ncbi.nlm.nih.gov/). And The targets of Pue were also searched through Pharmmapper (http://www.lilab-ecust.cn/pharmmapper/). Meanwhile, the targets of Pue were screened according to the principle of probability > 0 in SwissTargetPrediction (http://www.swisstargetprediction.ch/) database and Prob > 0 in Targetnet (http://targetnet.scbdd.com/) database, and the targets were supplemented in combination with literature in PubMed database. After merging, duplicate data were removed, and UniPort protein standardization database (https://www.uniprot.org/) was used to uniformly transform Pue-related targets.

Machine Learning

A total of 171 algorithmic combinations consisted of 13 machine learning methods were utilized in the above selected significant elements within the cohorts in our research, including XGBoost, glmBoost, NavieBayes, elastic network (Enet), generalised boosted regression modelling (GBM), least absolute shrinkage and selection operator (Lasso), partial least squares regression for glm (plsRglm), random forest, Ridge, supervised principal components (SuperPC), stepwise glm, support vector machine (SVM), LDA, neural network (NN) respectively (40, 41). Moreover, the average accuracy of each algorithmic combination across the whole training, test, and validation sets was considered as a criterion for judging the superiority of algorithmic combinations, in which the algorithmic combination with the highest average accuracy was selected into our subsequent elements' selection.
